# Vpr Promotes Macrophage-Dependent HIV-1 Infection of CD4^+^ T Lymphocytes

**DOI:** 10.1371/journal.ppat.1005054

**Published:** 2015-07-17

**Authors:** David R. Collins, Jay Lubow, Zana Lukic, Michael Mashiba, Kathleen L. Collins

**Affiliations:** 1 Department of Microbiology & Immunology, University of Michigan, Ann Arbor, Michigan, United States of America; 2 Department of Internal Medicine, University of Michigan, Ann Arbor, Michigan, United States of America; 3 Medical Scientist Training Program, University of Michigan, Ann Arbor, Michigan, United States of America; 4 Graduate Program in Immunology, University of Michigan, Ann Arbor, Michigan, United States of America; Fred Hutchinson Cancer Research Center, UNITED STATES

## Abstract

Vpr is a conserved primate lentiviral protein that promotes infection of T lymphocytes in vivo by an unknown mechanism. Here we demonstrate that Vpr and its cellular co-factor, DCAF1, are necessary for efficient cell-to-cell spread of HIV-1 from macrophages to CD4^+^ T lymphocytes when there is inadequate cell-free virus to support direct T lymphocyte infection. Remarkably, Vpr functioned to counteract a macrophage-specific intrinsic antiviral pathway that targeted Env-containing virions to LAMP1^+^ lysosomal compartments. This restriction of Env also impaired virological synapses formed through interactions between HIV-1 Env on infected macrophages and CD4 on T lymphocytes. Treatment of infected macrophages with exogenous interferon-alpha induced virion degradation and blocked synapse formation, overcoming the effects of Vpr. These results provide a mechanism that helps explain the in vivo requirement for Vpr and suggests that a macrophage-dependent stage of HIV-1 infection drives the evolutionary conservation of Vpr.

## Author Summary

Human immunodeficiency virus (HIV-1), the leading infectious killer worldwide, dysregulates the immune system primarily through infection and depletion of CD4^+^ T cells. The conserved HIV-1 Vpr protein has been previously shown to promote T cell infection and disease progression in an animal model; however, infection of primary CD4^+^ T cells in culture does not require Vpr, and its mechanism of action remains undefined. Here we show that Vpr promoted HIV-1 infection of CD4^+^ T cells by counteracting an antiviral restriction in infected primary macrophages. This restriction degraded HIV-1 in macrophages and impaired the formation of virological synapses–intercellular contact sites that facilitate efficient and immunoevasive viral transmission to T cells. Treatment of infected cells with the antiviral cytokine interferon-alpha induced this restriction even in the presence of Vpr, suggesting that Vpr prevents induction of an antiviral state in macrophages with consequences for viral spread to T cells. Our study provides mechanistic insight into the function of Vpr and the role of macrophage infection in HIV-1 pathogenesis, with implications for the development of improved treatment strategies.

## Introduction

HIV-1 Vpr is conserved in all primate lentiviruses. However, decades of research have not revealed a functional explanation for its evolutionary conservation. CD4^+^ T lymphocytes are the most abundant cellular target of HIV-1 in vivo and are widely regarded as the main drivers of viremia, persistence and progression to acquired immunodeficiency syndrome [1]. While Vpr enables robust T lymphocyte infection and rapid disease progression in vivo [2,3] and in ex vivo human lymphoid tissue [4], Vpr is dispensable and may actually be detrimental to HIV-1 replication in T lymphocytes in vitro [5–7]. Recent work using transformed cell lines has defined a molecular mechanism by which Vpr limits immune detection of HIV-1 through modulation of host cellular ubiquitin ligase pathways and activation of a cellular nuclease [8]. Vpr modulates these pathways at least in part through its interaction with its cellular co-factor DCAF1 (also known as VprBP) [9,10]. Vpr utilizes this pathway to counteract a macrophage-specific restriction of HIV-1 Env glycoprotein expression [11]. However, in T lymphocytes, there is no defect in Env expression in the absence of Vpr [11] and it remains unclear how Vpr enhances HIV-1 replication in CD4^+^ T lymphocytes in vivo [12,13].

In this study, we describe cell culture conditions in which HIV-1 infection of primary T lymphocytes depended entirely on contact-dependent spread from monocyte-derived macrophages (MDM); a mode of spread that evaded neutralization by some antibodies. Under these conditions, Vpr enhanced the formation of virological synapses (VS) between infected MDM and primary T lymphocytes. Mechanistic studies revealed that Vpr functioned to prevent an innate immune response that dramatically reduced HIV-1 Env expression, normal virion trafficking and VS formation in MDM-T lymphocyte co-cultures. The addition of exogenous interferon-α (IFN) effectively counteracted the ability of Vpr to promote spread from MDM to T lymphocytes. Our results highlight the importance of macrophages in HIV-1 pathogenesis and explain a requirement for Vpr in HIV-1 infection of T lymphocytes, providing a previously elusive explanation for Vpr’s strong evolutionary conservation.

## Results

### Efficient infection of primary CD4^+^ T lymphocytes requires contact-dependent HIV-1 spread from infected macrophages

**Fig 1:**
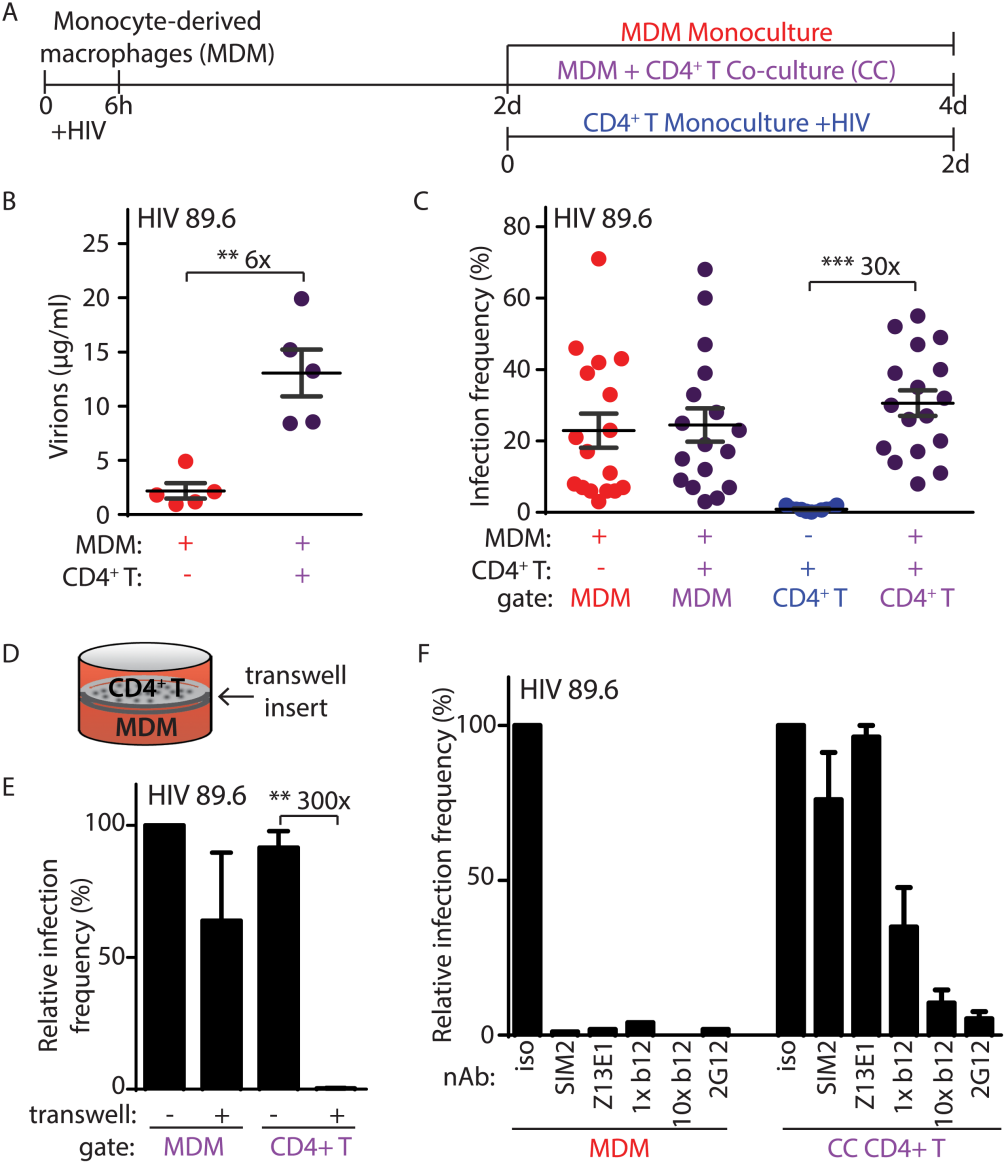
Efficient HIV-1 infection of T lymphocytes requires contact with infected macrophages. (A) Graphical outline of experimental setup depicting HIV-1 infection of MDM and co-cultivation with autologous, PHA-activated CD4^+^ T lymphocytes (CC) as detailed in Methods. (B) Summary graph of quantity of virions released into culture supernatant as measured by Gag CAp24 ELISA (n = 5 donors). (C) Summary graph of infected cell frequency in the indicated cultures as measured by flow cytometry (n = 11 donors for CD4^+^ T or 17 donors for MDM and CC). (D) Diagrammatic representation of virus-permeable transwell. (E) Summary graph of relative infected cell frequency in co-cultures prepared as shown in A in the presence or absence of transwell inserts (n = 4 donors). Infection frequency was determined by flow cytometry and values were normalized to MDM infection frequency without transwell insert. (F) Summary graph of relative infected cell frequency, as measured by flow cytometry and normalized to isotype (iso), in the indicated cultures prepared as shown in A. Neutralizing antibodies to HIV-1 Env gp120 (2G12, b12), gp41 (Z13E1) or CD4 (SIM.2) were added at the time of initial infection (MDM) or at the time of CD4^+^ T addition and co-cultivation (CC) at 1 μg/ml (1x) and/or 10 μg/ml (10x), as indicated. Error bars represent SEM. **p<0.01, ***p<0.001, student’s paired t-test. The color of the X axis label of each summary graph corresponds to the culture condition shown in part A.

To evaluate a role for Vpr in T lymphocyte infection that explained in vivo observations, we developed an assay to measure HIV-1 spread from primary MDM to autologous CD4^+^ T lymphocytes. As outlined in Fig 1A, we inoculated primary MDM with HIV-1 and allowed infection to establish for two days before co-cultivation with activated autologous CD4^+^ T lymphocytes for an additional two days to enable viral spread. MDM-T lymphocyte co-cultures produced an average of six-fold more HIV-1 than infected MDM alone, suggesting that co-cultivation resulted in efficient spread between MDM and T lymphocytes (Fig 1B).

To measure the frequency of infection in each cell type, we used flow cytometry to distinguish MDM from T lymphocytes by expression of surface markers and measured infection by intracellular Gag staining (S1A Fig). Detection of Gag^+^ cells was dependent on retroviral integration, demonstrating that our assay measures productive HIV-1 replication (S1B Fig). Although HIV-1 infects and depletes CD4^+^ T lymphocytes to cause acquired immunodeficiency syndrome in vivo, infection of primary CD4^+^ T lymphocytes by cell-free virus was inefficient in vitro after two days of continuous culture (Fig 1C and S1A Fig) using an inoculum comparable to the amount of virus present in MDM-T lymphocyte co-cultures. In comparison, co-cultivation of activated T lymphocytes with infected MDM increased T lymphocyte infection by thirty-fold (Fig 1C).

The capacity for MDM to efficiently infect autologous primary CD4^+^ T lymphocytes depended on direct cell-to-cell contact because infection was not detected when the cells were separated by a virus-permeable transwell insert (Fig 1D and 1E). Direct cell-to-cell transmission of HIV-1 across virological synapses between infected and target cells has been previously described and is known to be highly resistant to antibody neutralization [14,15]. Consistent with this mode of spread, we observed that MDM-dependent spread to autologous primary CD4^+^ T lymphocytes was highly resistant to a subset of neutralizing antibodies (b12, Z13E1 and SIM2) that inhibited greater than 95% of infection by free virus at the same antibody concentration (Fig 1F, compare left and right panels). In contrast, the monoclonal antibody 2G12, which is capable of disrupting cell-to-cell spread [16], was able to efficiently neutralize MDM-dependent T lymphocyte infection at 1 μg/ml (Fig 1F, right panel). Consistent with a previous report, a ten-fold higher concentration of b12 was also able to neutralize cell-to-cell spread (Fig 1F, right panel) [17].

Previous studies have demonstrated that uninfected dendritic cells and MDM can infect T lymphocytes through a “*trans”* mechanism in which virions bound to lectin receptors are transferred to T lymphocytes (S1C Fig) [18,19]. This contrasts with *“cis”* infection that requires HIV-1 replication in MDM. To determine the mode of infection that was active in our system, we used the protocol described in Fig 1A but substituted an HIV-1 molecular clone that can infect T lymphocytes but not MDM (NL4-3). Similar to HIV-1 89.6, cell-free HIV-1 NL4-3 did not efficiently infect primary T lymphocytes (S1D Fig). Consistent with previous reports [20], however, NL4-3 infected a high percentage of T lymphocytes upon spinoculation (S1D Fig). As expected, NL4-3 did not infect MDM (S1D Fig) and MDM treated with NL4-3 as outlined in Fig 1A did not spread infection to primary CD4^+^ T lymphocytes (S1D Fig). Thus, spread of infection from MDM to primary CD4^+^ T lymphocytes required productive HIV-1 replication in MDM under the conditions of our assay. In summary, efficient infection of primary CD4^+^ T lymphocytes required contact-dependent, neutralizing antibody-resistant, *cis*-mediated virus transfer from HIV-1 infected MDM.

### Vpr enables macrophage-dependent T lymphocyte infection

**Fig 2:**
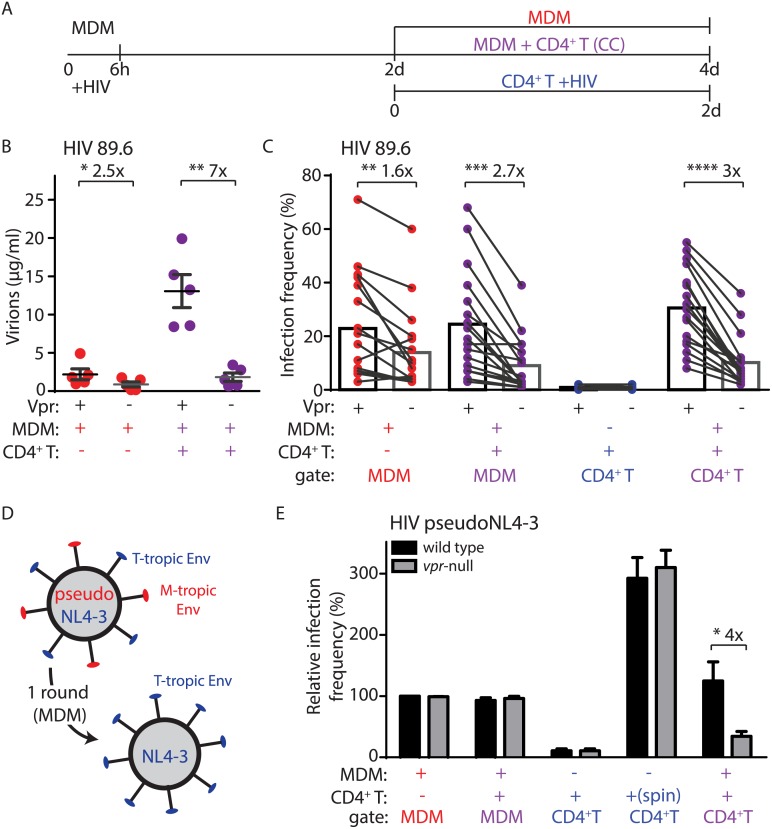
Vpr enhances macrophage-dependent infection of CD4^+^ T lymphocytes. (A) Graphical outline of experimental setup as in Fig 1A. (B) Summary graph of quantity of virions released into the supernatant of the indicated cultures after inoculation with wild type or *vpr*-null HIV-1 89.6 as indicated (n = 5 donors). (C) Summary graph of infected cell frequency in the indicated cultures (n = 11 donors for CD4^+^ T or 17 donors for MDM and CC) as measured by flow cytometry. (D) Diagram illustrating use of HIV-1 NL4-3 pseudotyped with YU-2 Env (red) to infect MDM for a single round and subsequent spread of wild type NL4-3 (blue) to T lymphocytes. (E) Summary graph of relative infected cell frequency in the indicated cell types after addition of HIV-1 YU-2 pseudo-NL4-3 as described in A. Infection frequency was measured by flow cytometry and normalized to infection frequency of wild type HIV-1 in MDM. The color of the X axis label of each summary graph corresponds to the culture condition shown in A, except that for “spin” condition, PHA-activated CD4^+^ primary T lymphocytes were centrifuged for 2500 RPM with 50μg HIV-1 NL4-3 in polybrene (n = 3 donors). Error bars represent SEM. *p<0.05, **p<0.01, ***p<0.001, student’s paired t-test.

The HIV-1 Vpr protein is necessary for optimal infection and spread in MDM cultures but can actually be detrimental to spread of infection in actively replicating cells due to its inhibitory effects on cell cycle progression [7,21,22]. Because CD4^+^ T lymphocytes are the main target of HIV-1 in vivo, Vpr’s role in HIV-1 infection and its evolutionary conservation across lentiviral species targeting a wide range of primates has remained enigmatic [23]. We hypothesized that the mode of spread we describe here, in which efficient T lymphocyte infection was dependent on infected MDM, might reveal a crucial role for Vpr in enabling efficient T lymphocyte infection. To address this, we co-cultivated activated primary CD4^+^ T lymphocytes with autologous MDM infected by HIV-1 89.6 containing or lacking Vpr (Fig 2A). Indeed, we observed a striking enhancement of infection by Vpr in our co-culture assay as measured by virion production (seven-fold, Fig 2B) and frequency of T lymphocyte infection (three-fold, Fig 2C). We observed similar results with the CCR5-tropic HIV-1 AD8 (three-fold, S2A Fig).

Because Vpr stimulates HIV-1 spread among MDM (Fig 2C) [11,24], it was possible that the stimulation of T lymphocyte infection we observed may result from an increase in the number of infected MDM that could amplify virus production. To address this, we measured spread of HIV-1 from infected MDM to T lymphocytes under conditions in which HIV-1 could only infect MDM for a single round and subsequent spreading infection could only occur in T lymphocytes. This was accomplished by using T-lymphotropic HIV-1 NL4-3 pseudotyped with macrophage-tropic YU-2 Env (Fig 2D). This virus utilizes YU-2 Env protein to efficiently infect MDM for one round of viral replication. However, *de novo* virions produced by the infected MDM express only NL4-3 Env and thus can only infect T lymphocytes. As previously reported [11], this virus initially infected MDM equally in the presence or absence of Vpr expression (Fig 2E). Remarkably, however, Vpr significantly enhanced spread of HIV-1 from infected MDM to T lymphocytes (four-fold, Fig 2E). In contrast, Vpr did not stimulate direct infection of primary T lymphocytes via spinoculation with cell-free virus (Fig 2E), or by spread of virus between T lymphocytes (S2B–S2E Fig), consistent with previous studies [5]. These data indicate that Vpr promotes the directional spread of HIV-1 from MDM to T lymphocytes and that this activity of Vpr is conserved in diverse HIV-1 isolates.

### Vpr-dependent HIV-1 spread from macrophages to T lymphocytes requires DCAF1

**Fig 3:**
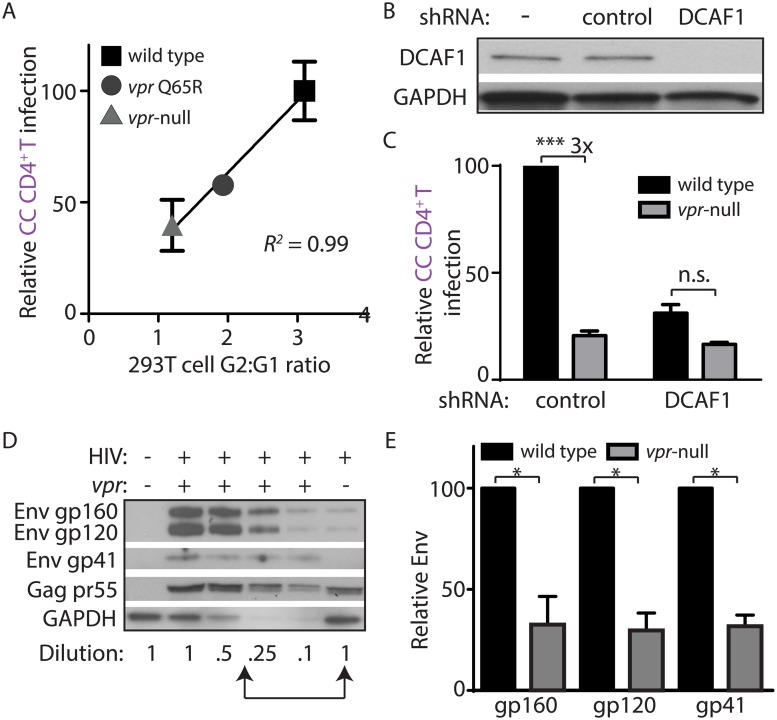
DCAF1 is required for Vpr-dependent HIV-1 spread from macrophages to CD4^+^ T lymphocytes. (A) Scatter-plot of Vpr-dependent cell cycle arrest in 293T cells (x-axis) versus relative infection frequency of CD4^+^ T lymphocytes co-cultured (“CC”) with infected MDM as outlined in Fig 2A. Infection frequency was assessed by flow cytometry and was normalized to that of wild type (y-axis). Best-fit curve from linear regression analysis, *R* ^*2*^ = 0.99 (n = 4 donors). (B) Immunoblot of DCAF1 and GAPDH in MDM seven days after transduction with lentivirus encoding shRNA targeting luciferase (“control”) or DCAF1. (C) Summary graph showing relative infection frequency of T lymphocytes co-cultured (“CC” as in Fig 2A) with MDM that had been treated with the indicated shRNA and infected with the indicated HIV-1 89.6 (n = 3 donors). (D) Immunoblot of HIV-1 89.6 Env and Gag in MDM-T lymphocyte co-culture whole-cell lysates diluted as indicated. Arrows denote lysates with comparable levels of Gag pr55 in the presence and absence of Vpr. (E) Summary graph of relative Env levels quantified by densitometry and normalized to Gag pr55 levels and to wild type (n = 4 donors). Error bars represent SEM. *p<0.05, ***p<0.001, “n.s.”p>0.05, student’s paired t-test.

Vpr interacts with the cellular protein DDB1-and-CUL4-associated factor 1 (DCAF1, also known as VprBP) to modulate ubiquitylation and proteasomal degradation pathways [9,25–27]. Recent work has demonstrated that DCAF1 is an essential co-factor for Vpr to evade the induction of a type I IFN response [8], and thereby counteract macrophage restriction of Env and virion production [11]. To determine whether this pathway was required for spread of HIV-1 from infected MDM to primary T lymphocytes, we employed the Vpr Q65R mutant of 89.6 that is deficient at interacting with DCAF1 and relatively defective at inducing DCAF1-dependent cell cycle arrest [11,28]. Using the co-culture assay described in Fig 2A, we found that Vpr Q65R was proportionally defective at enhancing HIV-1 spread from MDM to CD4^+^ T lymphocytes (Fig 3A). To more directly address the requirement of DCAF1 for Vpr-dependent spread, we silenced DCAF1 in infected MDM (Fig 3B) and co-cultured these cells with autologous T lymphocytes. Remarkably, we found that DCAF1 silencing abrogated the ability of Vpr to stimulate transmission of HIV-1 from MDM to CD4^+^ T lymphocytes (Fig 3C). While DCAF1 is required for Vpr to stabilize Env [11], its silencing also induces IFN in HeLa cells [8], raising the possibility that MDM silenced for DCAF1 produce IFN that may reduce T lymphocyte permissivity. To examine this, we used quantitative RT-PCR to measure IFN induction in MDM treated with control shRNA or shRNA directed against DCAF1. As shown in S3A Fig, there was no significant difference in *IFNA1 and MXI* induction between these two conditions, indicating that DCAF1 silencing does not stimulate an IFN response in MDM. To extend these results, we also examined whether soluble factors produced by MDM silenced for DCAF1 could contribute to reduced HIV-1 transmission. We found that conditioned medium from MDM silenced for DCAF1 did not suppress infection of activated primary T lymphocytes (S3B Fig). These results are consistent with a prior study that did not observe induction of IFN-stimulated genes in primary myeloid cells silenced for DCAF1 [29]. Collectively, these data demonstrate that Vpr requires DCAF1 to promote MDM-to-T lymphocyte spread of HIV-1 and that this requirement for DCAF1 is not due to soluble factors induced by DCAF1 silencing.

### Vpr prevents lysosomal targeting of Env-containing virions in macrophages

MDM infected by HIV-1 lacking Vpr restrict Env expression by accelerating lysosomal degradation of Env, and Vpr counteracts this pathway via a DCAF1-dependent mechanism [11]. Because DCAF1 was also required for Vpr-dependent MDM-T lymphocyte spread of HIV-1 (Fig 3A–3C), it is possible that restriction of Env expression leads to reduced spread from MDM to T lymphocytes. As a first step to address this possibility, we analyzed co-culture whole-cell lysates for steady-state Env expression by quantitative immunoblot in the presence or absence of Vpr (Fig 3D). Indeed, we observed a loss of Env gp160, gp120 and gp41 relative to the HIV-1 Gag precursor pr55 in the absence of Vpr in co-cultures (Fig 3E), similar to what was previously reported in HIV-1 infected MDM [11]. Remarkably, however, a similar analysis of HIV-1 protein expression from the non-adherent T lymphocyte fraction of the co-culture did not reveal a Vpr requirement for Env expression (S3C Fig), consistent with our model that Vpr counteracts an MDM-intrinsic restriction of Env.

Because Vpr and DCAF1 are required for Env stability and virion incorporation [11], we sought to address whether Vpr increases T lymphocyte infection by increasing virion infectivity. To examine this, we harvested virus from MDM infected with wild type or Vpr-null HIV-1 and used these MDM-derived virions to infect activated primary T lymphocytes via spinoculation. Consistent with our prior observations [11], we found that there was no significant difference in infection frequency when T lymphocytes were infected with equal mass amounts of cell-free virus collected from wild type and Vpr-null-infected MDM (S3D Fig). Thus, under the conditions of our assay, Vpr acts primarily by counteracting a cell-intrinsic pathway in MDM that restricts efficient transfer of virions to T lymphocytes rather than by increasing virion infectivity.

Because Vpr and DCAF1 cooperate to counteract induction of a type I IFN response [8,11], we also sought to determine whether reduced MDM-dependent T lymphocyte infection in the absence of Vpr may be mediated by soluble IFN produced by Vpr-null-infected MDM. To this end, we neutralized the type I IFN receptor (IFNAR2) at the time of co-culture, but still observed a Vpr requirement for T lymphocyte infection (S3E Fig). Additionally, pretreatment of T lymphocytes with conditioned supernatants from MDM infected in the presence or absence of Vpr did not block HIV-1 infection by spinoculation (S3F Fig). Thus, infection activates an intrinsic antiviral pathway in MDM that primarily acts to restrict viral spread rather than to release soluble antiviral factors that influence T lymphocyte permissivity.

**Fig 4:**
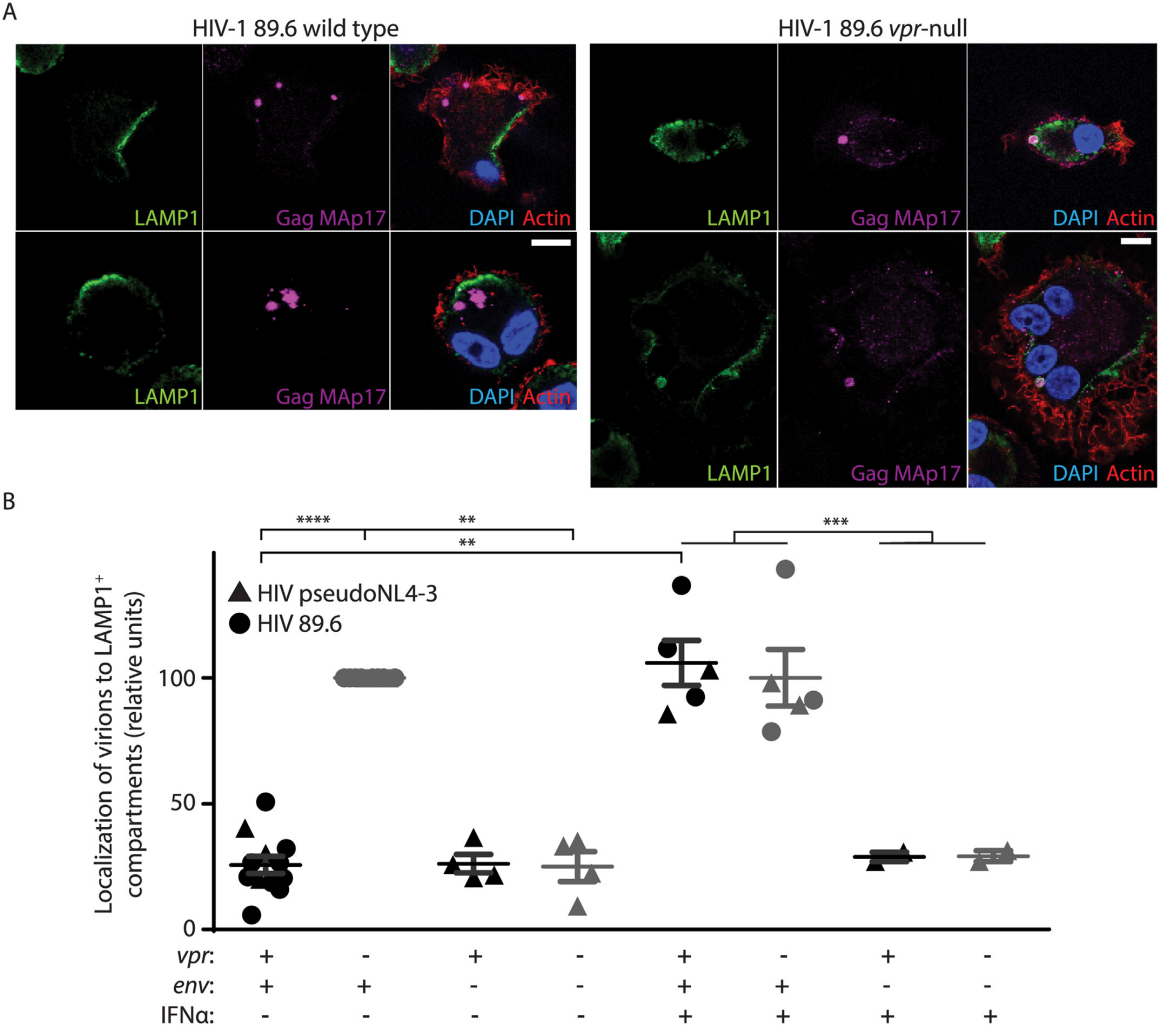
Vpr counteracts Env-dependent targeting of HIV-1 virions to lysosomes in macrophages. (A) Representative confocal micrographs depicting subcellular localization of lysosome marker LAMP1 (green) and HIV-1 Gag MAp17 (magenta) in MDM infected by wild type or Vpr-null HIV-1 89.6 for ten days. Merged images (right panels) include phalloidin staining of actin cytoskeleton (red) and DAPI staining of nuclei (blue). Scale bars (white) represent 10 μm. (B) Summary graph displaying the number of Gag (MAp17^+^) puncta co-localizing with LAMP1^+^ organelles. Results were normalized to the number of infected cells analyzed and to the *vpr*-null condition. Each point represents data from a separate donor and each symbol represents infection by a different HIV molecular clone as indicated. Where indicated, IFNα was added for the final two days of infection. In all conditions, lysosomal acidification was blocked with 20mM NH_4_Cl for the final 8 hours. **p<0.01, ***p<0.001, ****p<0.0001, student’s paired t-test.

To further characterize how viral spread is restricted, we sought to determine the mechanism by which MDM restricted efficient transfer of virions to T lymphocytes in the absence of Vpr. Our prior studies have demonstrated that: (1) Vpr prevents degradation of Env in lysosomes, (2) Env is required for Vpr-dependent changes in virion release, and (3) that there are significantly fewer cell-associated virions in MDM infected with Vpr-null HIV-1 based on immunoblot analysis of Gag p24 [11]. Thus, we hypothesized that in the absence of Vpr, Env-containing virions are targeted for lysosomal degradation in MDM. To test this, we examined the co-localization of mature virions (Gag MAp17^+^) with LAMP1, a marker of lysosomes. Because HIV-1-infected cells form syncytia, infected MDM are frequently multinucleated, which we also observed (Fig 4A). Remarkably, in the absence of Vpr, mature virions (magenta puncta in Fig 4A, right panels) frequently co-localized with LAMP1. In comparison, expression of Vpr reduced co-localization of mature virions with lysosomal markers (Fig 4A and 4B). In addition, we observed more virions present in LAMP1^+^ compartments when lysosomal acidification was blocked by NH_4_Cl treatment, but not when proteasomal degradation was inhibited by MG132 treatment, indicating that colocalization with LAMP1 represents bona fide lysosomal targeting that results in significant degradation (S4A Fig). We observed similar results when co-staining was performed with the lysosome marker LAMP2, but not with the endoplasmic reticulum membrane marker calnexin (S4B and S4C Fig). Remarkably, we also observed that lysosomal targeting of virions depended on expression of Env from the integrated provirus (Fig 4B and S4D Fig). These studies reveal that in the absence of HIV-1 Vpr, MDM restrict HIV-1 by targeting Env-containing virions for lysosomal degradation.

Because restriction of Env expression and virion release by wild type infected MDM is inducible by type I IFN [11], we treated MDM with exogenous IFN to assess its effects on virion localization. Interestingly, IFN stimulated lysosomal targeting of virions even in MDM expressing Vpr, but only if Env was present (Fig 4B), providing further support for the model that Vpr functions to prevent an IFN-inducible restriction of Env and Env-containing virions in MDM.

### Vpr increases Env-dependent virological synapse formation between macrophages and T lymphocytes

**Fig 5:**
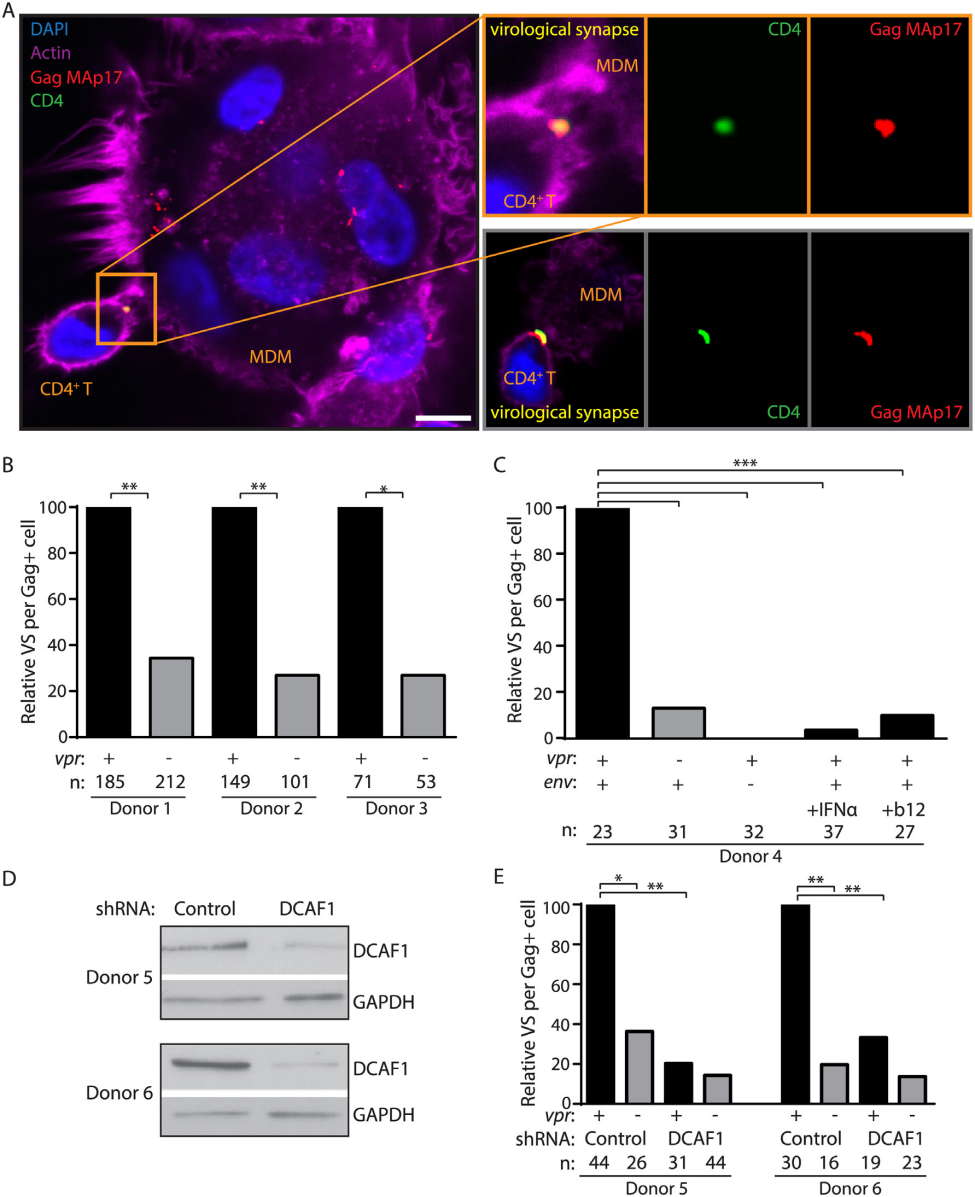
Vpr promotes Env-dependent virological synapse formation between macrophages and CD4^+^ T lymphocytes. (A) Representative confocal micrographs of MDM infected for seven days and briefly co-cultured with CD4^+^ T lymphocytes pre-stained for surface CD4. Co-localization between HIV-1 Gag MAp17 (red) in MDM and surface CD4 (green) on T lymphocytes is indicated as virological synapses (VS). Merged images include phalloidin staining of actin (magenta) and DAPI staining of nuclei (blue). Inset depicts magnified VS from same image (top) or from a different representative image (bottom). Scale bar (white) represents 10 μm. (B) Summary graph of relative VS observed per ‘n’ number of Gag^+^ MDM from three donors infected by wild type or *vpr*-null HIV-1 89.6. (C) Summary graph of relative VS per ‘n’ number of Gag^+^ MDM, as in B, of MDM infected with YU-2 Env-pseudotyped *env*-null 89.6 (third column), wild type 89.6-infected MDM treated for two days prior to co-culture with interferon-α (IFNα, fourth column) or treated with 10 μg/ml (10x) anti-Env gp120 neutralizing antibody b12 during co-cultivation with CD4^+^ T lymphocytes (final column), (D) Immunoblot of DCAF1 and GAPDH in MDM from two donors after silencing of DCAF1 as outlined in Methods. (E) Summary graph of relative VS per ‘n’ number of Gag^+^ MDM, as in B, of MDM treated with control or DCAF1-specific shRNA and infected with the indicated HIV-1 89.6. *p<0.05 **p<0.01 ***p<0.001, Fisher’s exact test.

Infection of T lymphocytes in our culture system occurs by direct cell-to-cell spread, which requires formation of transient VS between an infected cell and its target. Formation of VS requires interactions between HIV-1 Env on infected cells and CD4 on target cells [30]. Upon VS formation, high concentrations of mature virions localize to VS to mediate cell-to-cell spread [31]. Because Vpr rescues Env and Env-containing virions from lysosomal degradation, we hypothesized that Vpr would also enable the formation of VS in the co-culture system. To determine whether Vpr affects VS formation between MDM and primary T lymphocytes, we used laser-scanning confocal microscopy to visualize areas of co-localization between surface CD4 on T lymphocytes and mature virions in MDM. We pre-stained T lymphocytes with an anti-CD4 antibody (DK4003) that does not disrupt the ability of CD4 to bind Env, and co-cultured these cells with infected MDM briefly to allow formation of cellular contacts. We then washed away unbound cells and stained with an antibody against Gag MAp17 to visualize mature virions, as previously described [16,32,33]. Virological synapses were identified as regions of co-localization between CD4 (green puncta in Fig 5A) on T lymphocytes and mature Gag on MDM (red puncta in Fig 5A). We identified similar numbers of MDM infected with wild type and mutant virus, and infected MDM were frequently multi-nucleated syncytia (Fig 5A). However, we consistently observed significantly more VS per infected MDM in the presence of Vpr (Fig 5A and 5B). These results explain why spread of HIV-1 from MDM to T lymphocytes is dramatically enhanced by Vpr.

As has been shown for other types of cell-to-cell spread [30], we observed that VS between MDM and primary T lymphocytes did not form in the absence of de novo Env expression (YU-2 Env-pseudotyped HIV-1 89.*6env* ^*−*^) (Fig 5C). Furthermore, consistent with a previous report [17], VS formation was efficiently blocked by treating infected MDM with a high concentration (10 μg/ml) of the broadly-neutralizing anti-Env gp120 antibody b12 at the time of co-culture (Fig 5C). Thus, VS formation between HIV-1 infected MDM and primary T lymphocytes requires HIV-1 Env expression and is markedly enhanced by expression of Vpr in MDM.

Vpr enhances Env expression by counteracting a type I IFN-inducible restriction of Env expression [11] that targets Env-containing virions for lysosomal degradation (Fig 4). Therefore, we asked whether the addition of IFN to infected MDM affected VS formation with T lymphocytes. Indeed, we observed that IFN significantly reduced the number of VS detected per infected MDM even when Vpr was expressed (Fig 5C). Because Vpr and DCAF1 cooperate to counteract type I IFN induction, we also sought to determine whether DCAF1 is required for Vpr-dependent VS formation. To test this, we silenced DCAF1 in MDM from two donors (Fig 5D) and no longer observed significant Vpr-dependent VS formation with T lymphocytes (Fig 5E). In sum, these results are consistent with a model in which Vpr and DCAF1 cooperate to increase HIV-1 infection of T lymphocytes by counteracting a type I IFN-inducible restriction of Env-dependent VS formation in MDM that reduces efficient transfer of virions from MDM to autologous primary T lymphocytes.

## Discussion

Vpr is a highly conserved HIV-1 protein that is required for full pathogenesis in vivo by a mechanism that is poorly understood. Here we show that under conditions in which efficient CD4^+^ T lymphocyte infection required contact-dependent VS formation with infected MDM, Vpr promoted VS-mediated transmission of HIV-1. Moreover, we provide evidence that Vpr promoted infection by counteracting an IFN-inducible restriction of HIV-1 Env expression in MDM.

Although CD4^+^ T lymphocytes are the most abundant HIV-1-infected cell type in vivo and are responsible for much of its pathogenesis, T lymphocytes are relatively refractory to infection by cell-free HIV-1 in vitro. In contrast, we observed significantly more HIV-1 infection of activated primary CD4^+^ T lymphocytes when T lymphocytes were co-cultured with autologous infected MDM, despite similar amounts of free virus in the co-culture supernatant. These results are consistent with research from other investigators showing cell-to-cell spread is much more efficient than infection of T lymphocytes by cell-free virus [32,34]. We also observed that once low-level initial infection of T lymphocytes by cell-free virus was established, subsequent spread within the culture became highly efficient and Vpr-independent. Thus, in the in vitro co-culture system, Vpr and macrophages help the virus overcome a bottleneck to initial infection, accelerating infection of T lymphocytes. In this respect, the co-culture system recapitulated the in vivo requirement for Vpr for maximal T lymphocyte infection and provides a mechanism that helps explain its evolutionary conservation.

As reported by others [16,32], we demonstrate that HIV-1-infected MDM efficiently spread HIV-1 to T lymphocytes across Env-dependent VS, and that this mode of spread is resistant to neutralization by some antibodies. Furthermore, we show that productive infection of MDM was required for spread to T lymphocytes; passive *trans*-infection of T lymphocytes by uninfected MDM was not observed under the conditions of our assay. These results reveal a critical role for macrophage infection in maximal HIV-1 infection of T lymphocytes.

Our previous work indicates that Vpr increases MDM infection by preventing lysosomal degradation of Env and amplifying release of Env-containing virions [11]. We report herein that in the absence of Vpr, virions containing Env were targeted to macrophage lysosomes and fewer virions were localized to Env-dependent VS between MDM and T lymphocytes. Indeed, our results illustrate that Vpr from multiple HIV-1 isolates promoted efficient macrophage-dependent T lymphocyte infection by this mechanism. This conserved function of Vpr provides a mechanistic explanation for its evolutionary conservation.

Finally, we provide confirmatory evidence that Vpr prevents the activation of an innate immune restriction of HIV-1 in MDM. Vpr activates the SLX4 endonuclease complex through its adaptor protein, DCAF1, allowing HIV-1 to evade the induction of a type I IFN response [8]. This pathway is active in MDM and may explain how Vpr prevents macrophage-specific restriction of Env [11]. Consistent with this, we demonstrated that treatment of infected MDM with exogenous IFN increased Env-dependent lysosomal targeting of virions and impaired Env-dependent VS formation with T lymphocytes. While the involvement of DCAF1 and IFN in Vpr-dependent HIV-1 spread from MDM to T lymphocytes supports a potential role for SLX4-mediated immune evasion, this has not yet been directly demonstrated.

IFN has several well-documented antiviral effects and likely acts through multiple mechanisms to inhibit HIV-1 infection and spread. While we cannot exclude the possibility that IFN affects VS formation through additional mechanisms, our results suggest that the Env-dependent restriction observed in MDM in the absence of Vpr is inducible by exogenous IFN treatment. Whether the restriction observed in Vpr-null-HIV-1-infected MDM requires secreted IFN is an interesting possibility that requires further study. Restriction of HIV-1 by IFN is of particular interest in light of recent evidence that IFN treatment may shrink the HIV-1 reservoir [35,36]. Further elucidation of this pathway, including the mechanism by which HIV-1 is detected and the identity of the IFN-stimulated macrophage restriction factor are important areas for future investigation.

In sum, we report a novel role for Vpr in promoting VS-mediated HIV-1 infection of T lymphocytes by counteracting IFN-inducible restriction of Env in MDM. These results underscore the importance of macrophages in HIV-1 pathogenesis and antiviral immunity, and provide a compelling explanation for the *in vivo* function and evolutionary conservation of Vpr.

## Methods

### Antibodies

Antibodies to CAp24 (KC57-FITC, Beckman Coulter), CD3 (OKT3-Pacific Blue, BioLegend) and CD14 (HCD14-APC, BioLegend) were used for flow cytometry. Antibodies to the following proteins were used for immunoblot analysis: DCAF1 (11612-1-AP Proteintech), GAPDH (Santa Cruz Biotech), Gag pr55 (HIV-Ig), Env gp160/120, Env gp41, and Vpr (AIDS Reagent Program, Division of AIDS, NIAID, NIH: Catalog 288 from Dr. Michael Phelan [37], 11557 from Dr. Michael Zwick [38], 3951 from Dr. Jeffrey Kopp, and 3957 from NABI and NHLBI). Antibodies to the following proteins were used for microscopy: CD4 [DK4003 (Centre for AIDS Reagents, NIBSC, contributed by Dr. D Healey)], Gag MAp17 [4C9 (Centre for AIDS Reagents, NIBSC, contributed by Drs. R B Ferns and R S Tedder)], LAMP1 (H4A3), LAMP2 (H4B4) and calnexin (AF18) from Abcam. Secondary antibodies were FITC-conjugated goat anti-mouse IgG (H+L) and AlexaFluor 647-conjugated goat anti-mouse IgG2a (BD Biosciences). Neutralizing antibodies 2G12, b12, SIM.2, and Z13E1 (AIDS Reagent Program, Division of AIDS, NIAID, NIH: Catalog 1476 from Dr. Hermann Katinger [39], 2640 from Dr. Dennis Burton and Carlos Barbas [40], 723 from Dr. James E.K. Hildreth [41]) were used at a 1 μg/ml for neutralization studies at the time of co-culture, and b12 was used at 10 μg/ml to block VS formation and cell-to-cell spread. Anti-human IFNAR2 (MMHAR-2, PBL Assay Science) was used at 1 μg/ml for neutralization where indicated.

### Viral constructs

p89.6 and pNL4-3 were obtained through the AIDS Reagent Program, Division of AIDS, NIAID, NIH: catalogs 3552 and 114 from Dr. Ronald G. Collman and Dr. Malcolm Martin, respectively [42–44]. p89.6*vpr* ^-^, p89.*6env* ^*-*^, p89.6*vpr*Q65R, pNL4-3*env* ^*-*^, pNL4-3*vpr* ^-^, and pNL4-3*vpr* ^-^ *env* ^*-*^ were constructed as previously described [11]. pSIV3^+^, psPAX2, pAPM-1221 (shNC) and pDCAF-APM.1-3 (shDCAF1) were obtained from Dr. Jeremy Luban [45]. pYU-2*env* was obtained from Joseph Sodroski [46]. pAD8 and pAD8*vpr* ^*-*^ were obtained from Vicente Planelles [47].

### Virus preparation

Virus stocks were obtained by transfection of 293T cells with virus expression plasmids using polyethylenimine, as described [11,48]. Pseudotyped virus was produced by co-transfecting 293T cells with provirus and Env expression plasmid, as described [11]. Viral supernatants were collected at 48h and centrifuged at 1500 rpm to remove cell debris. Virus was stored at -80°C and quantified by CAp24 ELISA, as described [11].

### Cell isolation, HIV-1 infection and MDM-T lymphocyte co-culture

Leukocytes isolated from anonymous donors by apheresis were obtained from New York Blood Center Component Laboratory. Peripheral blood mononuclear cells (PBMC) were purified by Ficoll density gradient separation, as described [49]. CD14^+^ monocytes and CD4^+^ T lymphocytes were isolated as previously described [11]. Briefly, monocytes were isolated by positive selection with an EasySep magnetic sorting kit (StemCell Technologies). Monocyte-derived macrophages (MDM) were obtained by culturing monocytes in R10 [RPMI-1640 with 10% Certified endotoxin-low fetal bovine serum (Gibco, Invitrogen)], penicillin (10 Units/ml), streptomycin (10 μg/ml), L-glutamine (292 μg/ml), carrier-free M-CSF (50 ng/ml, R&D Systems) and GM-CSF (50 ng/ml R&D Systems) for seven days. MDM were incubated with 5 μg HIV-1 for six hours and cultured in fresh medium for two to four days. CD4^+^ T lymphocytes were isolated by CD8 negative selection (DynaBeads, Life Technologies), cultured in R10 for several days and activated with 5 μg/ml phytohaemagglutinin (PHA-L, Calbiochem) overnight before addition of 500 IU/ml recombinant human IL-2 (R&D Systems). T lymphocytes were infected with 5 μg or 50 μg HIV-1 by spinoculation at 2500 RPM for 2–3h with 8 μg/ml polybrene (Sigma) 72h following PHA stimulation, as described [49], or incubated with virus for two days, where indicated. For co-culture experiments, HIV-1-infected MDM were co-cultured with autologous CD4^+^ T lymphocytes 72 hours after PHA activation for two days. Infected T lymphocyte monocultures or co-cultures were maintained in R10 and IL-2 until analyzed. Where indicated, control cells were treated at the time of infection with 4 μM raltegravir (Selleck Chemical) to block retroviral integration.

### Flow cytometry

Surface staining for CD3 and CD14 was performed before fixation and intracellular staining for Gag CAp24 was performed as described previously [11,50]. Flow cytometric data was acquired using a FACSCanto instrument with FACSDiva collection software (BD) or a FACScan (BD, Cytek) with FlowJo software (TreeStar) and analyzed using FlowJo. Cell cycle analysis of 293T cells was performed previously [11]. Where indicated, cells were labeled with CMTMR fluorescent dye (Life Technologies) following the manufacturer’s protocol.

### Immunoblot

MDM or MDM-T lymphocyte co-cultures were lysed in Blue Loading Buffer (Cell Signaling), sonicated with a Misonix sonicator (Qsonica, LLC.) and clarified by centrifugation at 13000 RPM. Lysates were analyzed by SDS-PAGE immunoblot and protein levels were quantified using Adobe Photoshop as described [11,49].

### CAp24 ELISA

CAp24 ELISA was performed as previously described and quantitation of mass is based upon commercial standards (ViroGen) [11].

### RNAi

Short hairpin RNA-mediated knockdown of DCAF1 was performed as previously described [11,45]. Briefly, we spinoculated primary monocytes with VSV-G-pseudotyped SIV3^+^ for 2 hours with 10 μg/ml polybrene to allow Vpx-dependent downmodulation of SAMHD1. Cells were then incubated overnight in R10 with M-CSF (50 ng/ml) and GM-CSF (50 ng/ml) plus 20 μg VSV-G-pseudotyped lentivirus containing a shRNA cassette targeting luciferase (Control) or DCAF1. Following an overnight incubation, the cells were cultured for 3 days in fresh medium before addition of 10 μg/ml puromycin for 3 additional days prior to HIV-1 infection.

### Laser-scanning confocal microscopy (LSCM)

LSCM of MDM or MDM-T lymphocyte VS was performed as described previously [16,32], with modifications. Briefly, MDM were differentiated on Nunc Lab-Tek 4-well chambered borosilicate cover glass (Thermo Fisher). For VS visualization, autologous, PHA/IL-2-activated CD4^+^ T lymphocytes were pre-stained for surface CD4 for one hour with primary antibody plus 30 minutes with secondary antibody and co-cultured for four hours at room temperature with MDM before gentle washing with warm RPMI. For experiments using exogenous IFN, infected MDM were treated with 500 U/mL recombinant IFN-α (Calbiochem) two days before harvest. For LAMP1 staining, infected MDM were treated with 20 mM ammonium chloride for the final eight hours to prevent lysosomal acidification unless otherwise noted. Cells were fixed in 4% paraformaldehyde for one hour at room temperature and permeabilized with 0.1% saponin (Sigma) in 10% pooled human AB and goat sera for F_C_-receptor blocking for one hour at room temperature, and endogenous biotin was blocked using endogenous biotin-blocking kit (Life Technologies) before staining for Gag p18 and/or LAMP1 for one hour primary and 30 minutes secondary using the antibodies listed above. Actin cytoskeleton was visualized by Phalloidin-TRITC (Sigma) and nuclei were stained using DAPI (Fisher Scientific). Cells were preserved in ProLong Gold anti-fade (Life Technologies) and visualized on a Leica SPX5 inverted confocal microscope at the University of Michigan Microscopy and Image-Analysis Laboratory. Images of optical sections of approximately 1 μm depth were captured at 20X dry or 100X oil-immersion objective magnification. Images were processed using ImageJ (NIH) and co-localization was quantitated by automated spots analysis using Imaris (BitPlane). Each Gag MAp17^+^ puncta with signal 2-fold or greater above background based on a raltegravir-inhibited infected MDM control was identified in an automated manner, and fluorescence intensity in each channel was quantitated for each Gag^+^ spot. Co-localization was defined as the number of Gag^+^ spots that were also positive for LAMP1 or CD4 (VS) two-fold or greater above isotype staining controls, per Gag^+^ cell imaged.

### Quantitative RT-PCR

RNA was collected with RLN buffer (50 mM Tris-Cl, pH 8.0, 140 mM NaCl, 1.5 mM MgCl_2_, 0.5% (v/v) Nonidet-P-40) and spun at 300 x g for 2 minutes. Supernatant was transferred to a new microcentrifuge tube and resuspended in RLT buffer. RNA was isolated from MDMs using RNeasy Kit (Qiagen) with on-column DNase I digestion. RNA was reverse transcribed using iScript Advanced cDNA Synthesis Kit (Bio-Rad). cDNA was amplified with SsoAdvanced Universal SYBR Green Supermix (Bio-Rad) on an Applied Biosystems 7300 Real-Time PCR System using commercially available *IFNA1* primers (Prime PCR, qHsaCED0020782, Bio-Rad), synthesized *MX1* primers (Forward: 5’-TTG AGA CAA TCG TGA AAC AGC AA-3’, Reverse: 5’-TCC GTC ACG GTG TGT AGC ATA-3’), or with TaqMan Gene Expression Master Mix with *β-actin* primers and FAM-MGB probes (TaqMan Gene Expression, Hs99999903_m1, Life Technologies) (Applied Biosystems). Reactions were quantified using ABI Sequence Detection software compared to serial dilutions of a single-stranded DNA oligo spanning the *IFNA1* amplicon, *MX1* amplicon, or cDNA from mock-treated cells. Calculated copies from the no-RT controls were subtracted from the calculated copies of the cDNA samples, then normalized for input measured by *β-actin*.

### Accession numbers

Vpr (Q73369), DCAF1 (Q9Y4B6), LAMP1 (P11279), CD4 (P01730), Env (Q73372), Gag (Q73367), CD14 (P08571), CD3 (P07766), IFN-α (P01562), IFNAR2 (P48551), LAMP2 (P13473), Calnexin (P27824), MX1 (P20591).

## Supporting Information

S1 Fig Flow cytometric analysis of HIV-1 infection in macrophage-T lymphocyte co-cultures. (A) Representative flow cytometric dot plots illustrating segregation of CD14^+^ MDM from CD3^+^ T lymphocytes in co-cultures and subsequent assessment of HIV-1 infection by intracellular Gag CAp24 stain after treatment of the indicated cultures treated as shown in Fig 1A. (B) Representative flow cytometric dot plots as in A of MDM-CD4^+^ T lymphocyte co-cultures infected as shown in Fig 1A with the indicated amount of HIV 89.6 in the presence or absence of the integrase inhibitor raltegravir. (C) Diagram illustrating *trans*- and *cis*-infection of T lymphocytes. (D) Summary graph of infected cell frequency, as measured by flow cytometry, in the indicated cell type after addition of HIV-1 NL4-3 as described in Fig 1A. For “spin” condition, PHA activated CD4^+^ primary T lymphocytes were centrifuged for two hours at 2500 RPM with 50μg HIV-1 NL4-3 in polybrene (n = 4 donors). The color of the X axis label corresponds to the culture condition shown in Fig 1A.
(EPS)

S2 Fig Vpr promotes HIV-1 spread from macrophages to T lymphocytes but not between T lymphocytes. (A) Vpr stimulates spread of CCR5-tropic HIV-1 from MDM to autologous T lymphocytes. Summary graph of relative infection frequency of the indicated cells by wild type or *vpr*-null CCR5-tropic HIV-1 AD8, as measured by flow cytometry. Results were normalized to wild type-infected MDM (left panel) or to wild type-infected CD4+ T cells in co-culture (right panel). Data represent mean +/- SEM from four donors. The color of the X axis label corresponds to the culture condition shown in Fig 1A, where purple indicates that cells were co-cultured (“CC”). (B) Vpr does not stimulate cell-to-cell spread between activated CD4^+^ T lymphocytes. Summary graph of infection frequency (as measured by flow cytometry) in PHA-activated “Donor” CD4^+^ T lymphocytes that were spinoculated with the indicated virus, cultured alone for two days and then co-cultured for two additional days with autologous PHA-activated, uninfected “Recipient” CD4^+^ T lymphocytes that had been pre-labeled with CMTMR fluorescent dye. (C,D) There is a delay in achieving high infection frequency in PHA-activated CD4^+^ T lymphocytes inoculated with cell-free virus. Summary graphs of infection frequency (as measured by flow cytometry) at the indicated time points after cell-free inoculation with 50μg CAp24 of the indicated virus. (E) Loss of Vpr dependence of T lymphocyte infection rate in co-cultures over time. Summary graph of the Vpr-dependent increase in infection frequency (as measured by flow cytometry) observed in CD4^+^ T lymphocytes upon co-culture with autologous MDM for the indicated amounts of time. Data represent mean +/- SEM from two donors. **p<0.01, student’s paired t-test.
(EPS)

S3 Fig The effect of Vpr and DCAF1 on T lymphocyte infection requires a MDM cell-intrinsic process. (A) Summary graphs of *IFNA1* and *MX1* gene induction upon DCAF1 silencing, measured by quantitative RT-PCR as described in Methods. Each symbol represents a separate donor (n = 4). (B) Conditioned supernatant from MDM silenced for DCAF1 is not inhibitory to infection of CD4^+^ T lymphocytes. Summary graph of infection frequency in CD4^+^ T lymphocytes treated for 30 minutes with 0.5 ml conditioned supernatants (“supt”) from autologous MDM treated with the indicated shRNA, spinoculated with 10μg of wild type (black symbols) or *vpr*-null (gray symbols) HIV-1 89.6, cultured in the indicated supt for two days and analyzed by flow cytometry. Each donor is represented by a different symbol shape (n = 2). Data represent mean +/- SEM. (C) Vpr does not stimulate Env expression in CD4^+^ T lymphocytes co-cultured with infected MDM. Immunoblot showing expression of the indicated proteins in the non-adherent fraction of MDM-CD4^+^ T lymphocyte co-cultures infected by the indicated HIV-1. Where indicated, cultures were treated with raltegravir at the time of MDM infection (“d0”) or the time of co-culture (“d2”), lysed and diluted as indicated. Values represent densitometric ratios of the indicated proteins comparing wild type and *vpr*-null conditions. (D) Vpr does not significantly modulate virion infectivity. Summary graph of infection frequency in CD4^+^ T lymphocytes spinoculated with 1μg CAp24 of the indicated HIV-1 89.6 produced by MDM from three separate donors and analyzed at 2dpi by flow cytometry. Each donor is indicated by a different symbol (n = 3). Data represent mean +/- SEM. (E) IFNα suppresses MDM infection and MDM-to-T lymphocyte spread of wild type virus but IFNAR2 blockade does not rescue the Vpr-dependent defect in spread to of PHA-activated CD4^+^ T lymphocytes. Summary graph of infection frequency in the indicated cells treated with 500 U/ml IFNα and/or 1 μg/ml neutralizing antibody against IFNAR2 (where indicated) at the time of infection or co-culture. The color of the X axis label corresponds to the culture condition shown in Fig 1A where purple indicates that cells were co-cultured. (F) Conditioned supernatant from wild type and Vpr-null infected MDM does not affect infection frequency of PHA-activated CD4^+^ T lymphocytes. Summary graph of infection frequency in PHA-activated CD4^+^ T lymphocytes pretreated with 0.5 ml conditioned supernatants (“supt”) from MDM infected with the indicated virus. The pre-treated cells were subsequently spinoculated with 20 μg CAp24 and cultured for two days in the same supernatant they were pretreated with. Data represent mean +/- SEM from two donors.
(EPS)

S4 Fig Vpr increases lysosomal but not proteasomal or ER targeting of virions in MDM. (A) Summary graph displaying the number of Gag MAp17^+^ puncta co-localizing with LAMP1. Results were normalized to the number of Gag^+^ cells analyzed and to the *vpr*-null condition. Each point represents data from a separate donor and each symbol represents infection by a different HIV-1 molecular clone as indicated. Where indicated, cells were treated with 20 mM of NH_4_Cl or 2.5 μM of MG132 for eight hours prior to fixation. Data represent mean +/- SEM from the indicated number of donors. (B) Representative LSCM images of LAMP2 and Calnexin co-staining with Gag MAp17, DAPI and phalloidin staining of MDM. Scale bars (white) represent 10 μm. (C) Summary graph displaying the number of Gag (MAp17^+^) puncta co-localizing with the indicated organelle marker. Results were normalized to the number of infected cells analyzed and to the *vpr*-null condition. Each point represents data from a separate donor. We did not observe any colocalization between Gag MAp17 and Calnexin. (D) Summary table of raw data from individual donors as summarized in Fig 4B, including infection frequency as measured by flow cytometry (“%Gag+”), the number of Gag^+^ MDM (“Gag+ cells”) analyzed, the number of LAMP1^+^ Gag MAp17^+^ colocalized puncta (“Coloc. puncta”) observed, and the ratio of colocalized puncta to Gag^+^ MDM, for the conditions indicated in each heading.
(EPS)
